# Impact of Catheter Ablation for Atrial Fibrillation on Quality of Life

**DOI:** 10.3390/jcm11154541

**Published:** 2022-08-04

**Authors:** Ursula Rohrer, Martin Manninger, Andreas Zirlik, Daniel Scherr

**Affiliations:** Division of Cardiology, Department of Medicine, Medical University of Graz, 8036 Graz, Austria

**Keywords:** quality of life, atrial fibrillation, catheter ablation, pulmonary vein isolation

## Abstract

Atrial fibrillation is the most common sustained cardiac arrhythmia in adults. It is a complex arrhythmia leading to increased morbidity and mortality requiring thorough assessment and classification to guide therapy and to assess whether to pursue rate or rhythm control therapy. To obtain rhythm control, several strategies are available with different advantages and disadvantages concerning success rate and safety. Apart from antiarrhythmic drugs, catheter ablation is a well-established invasive therapy to treat atrial fibrillation. As quality of life is a very important factor to pursue rhythm control, several studies investigated on the specific impact of catheter ablation on quality of life. Catheter ablation shows a beneficial effect on quality of life in paroxysmal and persistent atrial fibrillation independent of the timepoint and strategy of catheter ablation.

## 1. Introduction

Atrial fibrillation (AF) is the most common arrhythmia in adults worldwide with a lifetime risk of more than 30%. It comes with increased morbidity and mortality and quality of life (QoL) is an important parameter in the context of AF and its therapeutic strategies [1]. AF-related QoL is often associated with functional status and exercise intolerance but can also be influenced by arrhythmia-related symptoms and signs, by the need for antiarrhythmic drugs (AAD) or oral anticoagulation (OAC), and by side effects/unwanted adverse effect of this medication. Furthermore, AF can influence patients’ QoL by an increased annual hospitalization rate and AF-related outcomes such as stroke, left ventricular (LV) dysfunction and heart failure, dementia, and depression. Concerning morbidity and mortality, cardio-embolic events as well as bleeding events due to OAC can lead to severe disabling situations with significantly decreased QoL [1].

The ESC guidelines on the management of atrial fibrillation recommend a holistic approach with the 4S-AF scheme: the QoL assessment is included in the part “symptoms” and “severity of burden”, while the other two “S” stand for “Stroke Risk” and “Substrate”.

This approach is feasible to characterize the arrhythmia and can guide the therapeutic strategy to improve clinical outcomes (any thromboembolic event, ischemic stroke, heart failure, acute coronary syndrome, significant coronary artery disease requiring coronary intervention and all-cause mortality) not only in the European but also in the Asian population [1,2,3,4].

Additionally, AF-specific QoL assessments and questionnaires can provide more detailed or complementary aspects such as concerns on a psychological level, adverse effects related to AAD or OAC and complementary information. The benefit of standardized assessments is the easy and valuable intercomparability over time to detect the effects and potential benefits or disadvantages of therapeutic strategy. Apart from characterization of the arrhythmia itself, these assessments can help guide the therapeutic strategy and may lead to early invasive rhythm control strategies [1].

In this mini-review, we aimed to describe the importance of QoL assessment to guide the therapy, the impact of catheter ablation on QoL at all stages whether asymptomatic or symptomatic and whether different timepoints or ablation strategies influence the effect on QoL.

## 2. Methods

PubMed, UptoDate, and ClinicalTrials.gov were searched for RCTs and relevant metanalyses that investigated on QoL and atrial fibrillation as well as its treatment strategies (last search update, 13 June 2022). In addition, the reference lists from initially identified articles were retrieved to avoid the exclusion of any relevant studies.

The following keywords were used: “atrial fibrillation”, “quality of life”, “catheter ablation”, and “pulmonary vein isolation”. The validation of the papers was carried out by an evaluation of the methodological quality of each eligible study.

## 3. Quality of Life in Atrial Fibrillation

Different assessments and questionnaires are used to find the right therapeutic strategy and to validate the therapy success.

### 3.1. Methods of Measuring QOL

The 2020 ESC AF guidelines recommend the assessment of QoL with the 4S-AF scheme, within the part focusing on “Symptom severity” in the initial assessment to characterize the arrhythmia (class IIa recommendation, level of evidence C) [1,4].

Within the 4S-AF scheme, the symptom severity and its impact on daily life and activity should be assessed with the European Heart Rhythm Association (EHRA) symptom score and additional specific questionnaires. The EHRA score categorizes arrhythmia-related symptoms and their effect on daily life analogously to the NYHA scale. EHRA score I implicates no arrhythmia-related symptoms, EHRA II describes mild symptoms but not affecting daily activity, patients with an EHRA III score have severe symptoms affecting daily activity, and EHRA score IV stands for disabling symptoms terminating daily activities [5].

The generally used QoL assessment may differ in validation and AF-specificity: well-established tests such as the Short Form Health Survey (SF36) and the EuroQol Five Dimensions Questionnaire (EQ5D) are extensively validated with a low specificity for AF-related QoL changes [5]. Large trials investigating QoL after AF ablation used AF-specific tests such as the “AF effect on Quality of Life Survey” (AFEQT) and the “Mayo AF Specific Symptom Inventory” (MAFSI) used in the CABANA trial [6], the “Quality of Life Questionnaire for Patients with AF” (AFQoL) used in the SARA study [7], the “University of Toronto Atrial Fibrillation Severity Scale” (AFSS) in the CTAF trial [8], the “Arrhythmia Specific Questionnaire in Tachycardia and Arrhythmia” (ASTA) used in MANTRA-PAF [9], and the “Arrhythmia-Related Symptom Checklist” (SCL) used in several trials and studies on AF-related QoL (see Table 1). The European Heart Rhythm Association (EHRA) score and the Canadian Cardiovascular Society Severity of Atrial Fibrillation Scale (CCS-SAF) may help to quickly assess the symptom burden on daily life and physical activity for a general characterization [5].

**Table 1:** Overview of QoL questionnaires.

Name	Specifications	Studies Used	AF Specific	Validated
Short Form Health Survey (SF-36)	Eight categories, scoring from 0 to 100 (vitality, physical functioning, bodily pain, general health perceptions, physical role functioning, emotional role functioning, social role functioning, mental health). Categories can be summarized in 2 groups: “physical component summary” (PCS) and “mental component summary” (MCS).	[10], CABANA trial [6], MANTRA PAF [9], A4 study [11], ThermoCool AF Trial [12,13], CAPTAF [14]	No	Yes
EuroQol Five Dimension Questionnaire (EQ5D)	Five dimensions of health state: mobility, self-care, usual activities, pain/discomfort, anxiety/depression. Can be used to analyze cost-effectiveness.	CABANA trial [6], MANTRA PAF [9], RAAFT2 [15]	no	yes
AF effect on Quality-of-Life Survey (AFEQT)	20 AF specific questions regarding symptoms, general daily activities, and treatment concerns	CABANA trial [6]	yes	yes
Mayo AF-Specific Symptom Inventory (MAFSI)	Evaluation of severity and frequency of AF related symptoms (10 categories)	CABANA trial [6]	Yes	yes
Duke Activity Status Index (DASI)	Assessment of functional capacity in cardiovascular diseases with 12 questions (0 to 58.2 points)	CABANA trial [6]	No	yes
Quality of Life Questionnaire for Patients with AF (AFQoL)	18 questions analyzing psychological, physical, and sexual aspects of daily life	SARA study [7]	yes	yes
University of Toronto Atrial Fibrillation Severity Scale (AFSS)	10 categories to assess symptom burden and severity	CTAF trial [8] CABANA trial [6]	Yes	yes
Arrhythmia-Specific Questionnaire in Tachycardia and Arrhythmia (ASTA)	Assessment of symptom and arrhythmia burden and 10 symptoms scored from 1 to 4	MANTRA-PAF [9]	yes	yes
Arrhythmia-Related Symptom Checklist (SCL)	16 questions regarding symptom burden and severity of AF	A4 study [11], ThermoCool AF Trial [12,13]	yes	yes
Minnesota Living with Heart Failure (MLwHF) questionnaire	2 equally scored items (physical and emotional dimension); total scores	PABA-CHF [16]	No	yes

Another part of the initial characterization is the duration of the arrhythmia which is usually classified as first diagnosed, paroxysmal, persistent, long-standing persistent, and permanent [1]. This classification is well established but may not entirely help to quantify the patients’ symptom burden. Upcoming investigations on AF and cardiac implantable electronic devices (CIED), as well as the possibility of more thorough rhythm monitoring with implantable loop recorders or wearable devices such as smartwatches or smartphone apps, may help in better assessment of AF burden and provide important symptom-rhythm correlations. A retrospective analysis of 798 patients with dual-chamber pacemakers from three trials identified a cut-off of more than two hours daily AF burden to have a negative impact on QoL compared to patients with CIEDs without AF [17].

After this characterization, a therapeutic strategy of rate or rhythm control will be determined in consultation with the patient, in a shared decision-based approach. According to the ESC AF guidelines, rhythm control therapy is recommended for symptomatic AF patients to improve symptoms and QoL (class I recommendation, level of evidence LoE A) [1]. However, this recommendation is based on X trials demonstrating that a medication-based rhythm control produces only minimally higher QoL improvement [18] and is associated with a significantly increased risk of adverse events in a meta-analysis of 25 randomized trials compared to medical rate control [19]. Apart from medical rhythm control, pulmonary vein isolation-based catheter ablation for AF is a well-established first- or second-line therapy depending on each patient’s specific characteristics in symptomatic patients [1].

According to the 2020 ESC guidelines for AF, catheter ablation has a class IIa recommendation (LoE B) as first-line therapy and a class I recommendation (LoE A) as second-line therapy after failed drug therapy in symptomatic paroxysmal AF. In patients with symptomatic persistent AF and without major risk factors for AF recurrence, catheter ablation has a class IIb recommendation (LoE C) as first-line and a class I recommendation (LoE A, LoE B if major risk factors for recurrence are present) as second-line therapy. In patients with paroxysmal or persistent AF and heart failure with reduced ejection fraction (HFrEF), catheter ablation is recommended as first-line therapy (class I recommendation, LoE B) to enable reverse left ventricular (LV) remodeling in suspected tachy-cardiomyopathy. Catheter ablation as second-line therapy still has a class IIa recommendation (LoE B) to reduce hospitalization and to improve survival in HFrEF patients without suspected tachy-cardiomyopathy. While the adopted first-line strategy can differ according to the patient’s choice and early catheter ablation can be performed without AAD failure [1].

### 3.2. Impact on QoL Comparing AAD to Catheter Ablation in Paroxysmal AF

Rhythm control strategies include classic antiarrhythmic drugs (AAD), pulmonary vein isolation (PVI), and as a usually last line or bailout strategy cardiac pacing and consecutive atrioventricular (AV)-node ablation. Several trials have investigated the impact of AAD vs. PVI on the impact of quality of life (see Table 1).

In 2005, a small randomized multicenter study including 70 symptomatic AF patients (“Radiofrequency Ablation vs. Antiarrhythmic Drugs as First-line Treatment of Symptomatic Atrial Fibrillation”) compared catheter ablation vs. AAD as a first-line therapy. The study cohort consisted mainly of patients with paroxysmal and a few patients with persistent AF. The data showed a superior effectiveness (*p* < 0.001) on arrhythmia freedom with an improvement in QoL assessed with the SF-36 questionnaire. This study focused on the feasibility of early catheter ablation and was not powered to prove the superiority of PVI as a first-line therapy. AF-specific questionnaires were not used in this study [10].

A few years after, the A4 study [11] was published with a similar setup: in a prospective multicenter RCT, catheter ablation was compared to various AAD (amiodarone, quinidine, disopyramide, flecainide, propafenone, cibenzoline, dofetilide, and sotalol were allowed alone or in combination) in patients with paroxysmal atrial fibrillation as second-line therapy. After a follow-up duration of 12 months, both groups showed a reduced AF burden with a benefit of PVI compared to AAD use (*p* = 0.0001). Within the QoL questionnaires, there was a statistically significant benefit of PVI on QoL assessed with the SF36 in several aspects (*p* = 0.01) as well as in symptom severity (*p* = 0.001) with no difference in symptom frequency (*p* = 0.10). The authors stated that freedom of AF was only achieved in 23–34% of patients with an AAD and suggest an early invasive strategy after one failed AAD as a conclusion of this data [11].

A multicenter RCT (ThermoCool AF trial) compared the safety and effectiveness of PVI after the failure of at least one AAD to the continuation of AAD (class I, III, or AV blocker), in a 2:1 ratio (106 patients underwent catheter ablation vs. 61 patients on AAD). Comparing the changes in QoL assessed with different questionnaires, the improvement in SF-36 physical and SF-36 mental summary scores was significantly higher after catheter ablation (*p* < 0.001). As in the A4 study, patients had lower baseline symptom severity scores when undergoing catheter ablation compared to AAD therapy. The improvement in the symptom severity score was higher in the ablation group (*p* < 0.001) comparable to the A4 study but opposed to results in the A4 study where there was no impact on the symptom frequency, the ThermoCool AF ablation cohort showed a decrease in symptom frequency after ablation (*p* < 0.001). This study also showed the effectiveness of catheter ablation on arrhythmia freedom (66% vs. 16% in the AAD group after 12 months) and the study authors suggest a benefit of early catheter ablation at least after AAD failure for an effective symptom control and QoL improvement. A multivariate analysis showed an interesting result: being randomized to catheter ablation and arrhythmia freedom showed the strongest association with QoL improvement from baseline to end of follow-up [12,13].

Following the positive results of the above-described trials that confirmed catheter ablation as an effective second-line therapy, the “Radiofrequency Ablation vs. Antiarrhythmic Drugs as First-Line Treatment of Paroxysmal Atrial Fibrillation” (RAAFT-2) trial investigated catheter ablation as a first-line therapy. In this study, the EQ5D score was used, which is a well-validated QoL score with the disadvantage of not being AF specific. The QoL improvement did not show a significant difference between both groups in this study, although the cohort randomized to catheter ablation experienced a lower rate of recurrence of atrial arrhythmia (54.5% versus 72.1% in the AAD group, *p* = 0.02) [15].

In the MANTRA PAF trial investigating radiofrequency (RF)-ablation vs. AAD as first-line therapy, both groups showed significant QoL improvement after initiation of therapy. At 24 months follow-up, a higher proportion of patients after ablation showed arrhythmia freedom (*p* = 0.004) in the 7-day Holter-ECG. A result that was associated with improved QoL in earlier studies, while MANTRA PAF (Medical Antiarrhythmic Treatment or Radiofrequency Ablation in Paroxysmal Atrial Fibrillation) patients randomized to radiofrequency-PVI showed only a little better QoL improvement with a focused improvement in physical scales (SF-36) in the EQ Visual Analogue Scale compared to patients on AAD [9,20].

Recent studies with higher patient numbers showed promising results regarding freedom of AF after catheter ablation, while the significance of QoL improvement through catheter ablation remains unclear. In 2019, the CAPTAF trial (Catheter Ablation compared with Pharmacological Therapy for Atrial Fibrillation) recruited 155 patients after treatment failure with AAD. Arrhythmia-free survival was assessed using implantable cardiac monitors and QOL was assessed using the SF-36 questionnaire. In contrast to earlier trials that performed radiofrequency ablation, the trial allowed cryoablation and additional RF lines at the physicians’ discretion. The trial resulted in a significant improvement of QoL assessed with the SF-36 (*p* = 0.003), an improvement of the EHRA score (*p* = 0.003), and a reduction in AF burden (*p* = 0.03) favoring catheter ablation while there was no difference in arrhythmia freedom between the two therapeutic strategies (*p* = 0.27) [14].

In the same year of publication of the data from the CAPTAF trial, the biggest randomized trial so far comparing AAD to catheter ablation in symptomatic AF patients was published. The CABANA trial (The Catheter Ablation vs. Antiarrhythmic Drug Therapy for Atrial Fibrillation (CABANA) included 2204 patients. The conservative cohort was treated with AAD for rhythm or rate control and the invasively treated cohort was scheduled for pulmonary vein isolation with additional lesions if needed. The goal of the study was to determine the superiority of catheter ablation in terms of QoL improvement. The cohort differed from former studies when it comes to baseline characteristics, as trials before focused on PAF patients. In the CABANA trial, the percentage was well-balanced between PAF and persAF patients (43% PAF, 57% persAF). In all assessed QoL scores and surveys, catheter ablation led to a significantly higher QoL improvement compared to AAD therapy with persisting effects even after 60 months of follow-up [6] (see Table 2).

The indication for PVI in paroxysmal AF is clearly defined and recommended in the current guidelines for AF management, compared to a more cautious recommendation for patients in persistent or even longstanding persistent AF, where the arrhythmia is already further progressed. In persAF, the substrate is more complex and freedom of AF after ablation is decreased compared to ablation in PAF patients. Data from randomized trials in paroxysmal AF suggested a correlation of arrhythmia freedom or reduction in burden with QoL. Looking into the lower success rate in persAF, the impact on QoL was material for further investigations (see Table 3).

**Table 3:** Studies concerning the impact of catheter ablation on QoL in persistent AF (CFAE: complex fractionated atrial electrograms, CRT: cardiac resynchronization therapy, LSP: long-standing persistent atrial fibrillation).

Name of Study	Study Design	Setup	Pat.	QoL Assessment	QoL Result
PABA-CHF trial, M.N. Khan, 2009 [16]	Prospective, multicenter, RCT	PVI vs. AV-node ablation and CRT-Implantation (persAF)	41	MLwHF	Favors catheter ablation (*p* < 0.001)
SARA study, L. Mont et al., 2014 [7]	Prospective, multicenter, RCT	RF-PVI vs. AAD, (PersAF)	146	AF-QoL questionnaire	No difference between groups (*p* = n.s.)
V. Bulkova et al., 2014 [21]	Prospective, multicenter, RCT	Catheter ablation in PAF vs. LSP-AF	387	EQ-5D	Better improvement in LSP-AF than in PAF (*p* = 0.03), improvement in both groups
S. Mohanty et al., 2014 [22]	Single-center, single arm prospective study	PVI+ CFAE + non-PV-trigger in asymptomatic LSP-AF	61	SF-36	No control group; QoL improvement over time
STAR AF, R. Mantovan et al., 2013 [23,24]	Prospective, multicenter, RCT	CFAE vs. PVI vs. CFAE + PVI combined in PAF and persAF	100	SF-36	No difference between groups, significant improvement (*p* < 0001)

### 3.3. Impact on QoL through Catheter Ablation in Persistent AF

A comparison of AAD versus catheter ablation did not prove a significantly different impact on QoL in patients with persAF. While low procedure-related complication rates compared to a high rate of AAD side effects might be reasons to choose an invasive rhythm control approach [7]. Even though guidelines remain restrictive with their recommendations on PVI in persistent AF [1], long-standing persistent AF(LSP-AF) patients seem to benefit more from an invasive strategy compared to PAF patients when looking into QoL improvement after catheter ablation. This improvement might be explained by a more profoundly reduced QoL at baseline as there is more way to improve. Moreover, younger patients and patients with a shorter history of AF seem to benefit more [21].

Further progression in arrhythmia does not always come with a higher symptom burden and patients with a long-lasting medical history of atrial fibrillation may adapt to their reduced physical ability which might explain that patients with a shorter history of AF experience a bigger QoL improvement when free of AF. To exclude subconscious adaptation to living with suboptimal physical capacity, the effect of QoL through rhythm control can be assessed after a successful cardioversion to differentiate whether there might be an improvement in sinus rhythm or not. While the data suggest that electrical cardioversion needs to be part of a long-term strategy including catheter ablation [25].

Whether no improvement of symptoms through rhythm control is detectable or patients are asymptomatic in the first place, guidelines do not recommend PVI in these individuals as long as no other criteria such as LV dysfunction are present [1,5]. A subconscious adaption to an arrhythmia-related reduced physical capacity needs to be excluded before being classified as asymptomatic. This again can be captured by a thorough characterization of arrhythmia, QoL questionnaires, and optionally a cardioversion trial to detect a beneficial effect of rhythm control, if reasonable. Specific situations can lead to an early invasive strategy, in line with the AF guidelines, patients with HFrEF and AF will be scheduled early for ablation.

In patients with AF and coexistent heart failure tachy-cardiomyopathy (tachyCMP) needs to be excluded. If tachyCMP is suspected, PVI should be performed early. Even if HF has another etiology, tachycardic conducted AF can lead to cardiac decompensation. Whether catheter ablation is not feasible or not successful, AV-node (AVN) ablation and biventricular pacing may be a bailout strategy in this specific cohort of patients. The PABA-CHF [16] trial compared whether PVI or AVN-ablation and biventricular pacing have a greater impact on QoL improvement, resulting in a greater QoL improvement through PVI in HF patients [16]. In the coexistence of AF and HF, catheter ablation can result in improvement of QoL and reverse LV remodeling, as well as reduced HF hospitalization and mortality [26].

Whether subconscious adaption, as well as coexisting HF, is excluded, there is no clear recommendation for catheter ablation in asymptomatic persAF cases [1]. Data from a small non-randomized, non-controlled trial provide hints that a successful procedure may still improve exercise performance and QoL even in asymptomatic LSP-AF patients: While the progression of the arrhythmia comes with a more complex arrhythmogenic substrate, AF was treated with an isolation of pulmonary veins as well as ablation of complex fractionated atrial electrograms (CFAE) and non-pulmonary vein triggers [22].

### 3.4. Impact of Ablation Strategy on QoL

The rising number of patients in persistent AF with already existing structural changes suggesting a more complex substrate and the not yet sufficient and satisfactory success rates open the debate about more complex ablations besides pulmonary vein isolation. The routine ablation of non-PV triggers or CFAE is not established for now and remains at the electrophysiologists’ discretion: the HRS/EHRA/ECAS/APHRS/SOLAECE expert consensus statement on catheter and surgical ablation of atrial fibrillation recommend that a CFAE ablation may be considered (IIb, LoE B-R) in persAF or LSP-AF but it is not recommended in PAF patients. Concerning non-PV triggers, high-dose isoproterenol may be administered during the procedure to detect and further ablate non-PV triggers independent of arrhythmia progression with a class IIb recommendation (LoE C-LD) [5].

The STAR-AF (The Substrate and Trigger Ablation for Reduction of Atrial Fibrillation) trial [23] compared ablation of CFAE, PVI only to a combined approach. The goal was to investigate a treatment strategy for patients with a high AF burden whether classified as paroxysmal or persistent. The ablation strategies showed very different outcomes concerning arrhythmia freedom: PVI only resulted in 48% of patients free of atrial arrhythmia, CFAE in 29%, and the combined approach in 74%. While the difference in arrhythmia freedom was significant (*p* = 0.004) there was no difference in impact on QoL. Patients with a recurring high arrhythmia burden showed a negative QoL affection. However, these results are hypothesis-generating since the trial was performed with low patient numbers and without continuous AF follow-up [23].

The subsequent STAR AF II trial showed a significantly shorter procedure time in PVI-only procedures compared to PVI plus additional non-PV trigger ablation (*p* < 0.001). Concerning arrhythmia freedom, the PVI-only cohort had no recurrence in 59%, the PVI and CFAE group in 49%, and the PVI and linear ablation in 46% (*p* = 0.15). This trial did not investigate QoL [27].

### 3.5. Impact of Post Ablation Recurrences on QoL

Results from various trials suggest an association of successful catheter ablation and arrhythmia freedom with QoL improvement [6,9,10,11,12,13,14,21], while only newer studies provide continuous rhythm follow-up with event recorders and can capture symptomatic as well as asymptomatic recurrences. Studies focusing specifically on the impact of the rhythm outcome after catheter ablation on QoL state that patients free of AF recurrence experience a greater improvement in functional status with no difference in QoL. There seems to be a difference in whether QoL is assessed by a general questionnaire compared to specific AF-related questionnaires [28,29]. Compared to symptomatic recurrences, silent AF recurrences do not seem to impact the physical component of QoL assessments [30].

## 4. Conclusions

Quality of life needs to be assessed at the first presentation with atrial fibrillation to characterize the arrhythmia according to the current ESC guidelines for the management of atrial fibrillation. The arrhythmic burden of AF seems to have a good correlation with QoL and helps to guide the therapeutic strategy in the first place while reduced QoL is a good indicator for early and efficient therapy in paroxysmal as well as persistent and long-standing persistent AF. As guidelines still preferably recommend antiarrhythmic drugs as a first-line therapy, data suggest that early catheter ablation leads to improved QoL and functional status compared to AAD. Efficacy and low procedure-related complication rates of catheter ablation compared to a high rate of AAD-associated side effects are good reasons to indicate catheter ablation at an early stage of AF. Even in patients without symptoms, catheter ablation seems to have beneficial effects on functional status and QoL as a long history of arrhythmia can lead to a subconscious adaption.

In the specific cohort of patients with AF and systolic heart failure, catheter ablation seems to be superior to a pace-and-ablate strategy and may help to reduce morbidity and mortality additionally to a positive impact on QoL.

The evidence for ablating non-PV triggers and CFAEs still remains scarce and additional ablation strategies on top of the well-established PVI have weak recommendations in expert consensus statements [5]. Substrate modification via catheter ablation such as targeting rotational activities or atrial scar tissue identified by cardiac MRI has been demonstrated to be without clinical benefit in large, randomized trials [31]. These strategies need to be investigated further to improve success rates in persAF and LSP-AF cases as success rates with PVI only are insufficient.

Nonetheless, maintenance of sinus rhythm after catheter ablation does not equal a QoL improvement. A recurrence does not seem to have a negative impact on QoL, as long as the arrhythmia burden remains low and/or the recurrences are silent and only detected by a thorough rhythm follow-up by event recorders or implantable devices.

Summarized, catheter ablation for atrial fibrillation helps to improve quality of life regardless of being first- or second-line therapy at any stage of arrhythmia.

## Figures and Tables

**Table 2:** Studies concerning the impact of catheter ablation on QoL in paroxysmal AF (RF-PVI: radiofrequency-pulmonary vein isolation, AAD: antiarrhythmic drug, RCT: randomized controlled trial, PAF: paroxysmal atrial fibrillation, persAF: persistent atrial fibrillation).

Name of Study	Study Design	Setup	Pat.	QoL Assessment	QoL Improvement after Catheter Ablation
O.M. Wazni et al. 2005 [10]	Prospective, multicenter RCT	RF-PVI vs. AAD—first line (PAF; single persAF)	70	SF-36	Better in 5/8 categories (*p* < 0.05)
A4 study, P. Jais et al. 2008 [11]	Prospective, multicenter RCT	RF-PVI + extra lines vs. AAD—second line (PAF)	112	SF-36, SCL	PCS and MCS better (*p* = 0.01) Symptom and severity of AF improved (*p* = 0.001)
ThermoCool AF Trial, D.J. Wilber et al., M.R. Reynolds, 2010 [12,13]	Prospective, multicenter, RCT	RF-PVI vs. AAD—second line (PAF)	167	SF-36, SCL	PCS, MCS, symptom and severity of AF improved (*p* < 0.001)
RAAFT2, C.A. Morillo et al. 2014 [15]	Prospective, multicenter, RCT	RF-PVI vs. AAD—first line, (PAF)	127	EQ-5D	All dimensions improved, not significant between groups (*p* = n.s.)
MANTRA PAF—subanalysis, H. Walfridsson 2014 [9]	Prospective, multicenter, RCT	RF-PVI as first line vs. AAD, (PAF)	294	SF-36, EQ-5D, ASTA	Better in 4/8 categories of SF-36 (*p* < 0.05), better in EQ-5D (*p* = 0.018), no difference in ASTA between groups (*p* = 0.30)
CAPTAF, C. Blomström-Lundqvist, 2019 [14]	Prospective, multicenter, RCT	RF-PVI as second line vs. AAD, (PAF)	155	SF-36	Significantly better (*p* = 0.003)
CABANA, D.B. Mark, 2019 [6]	Prospective, multicenter, RCT	RF-PVI as second line vs. AAD, (PAF)	2204	SF-36, EQ-5D, AFEQT, MAFSI, DASI, AFSS	Improvement in all questionnaires (*p* < 0.001)

## References

[1] Hindricks G., Potpara T., Dagres N., Arbelo E., Bax J.J., Blomstrom-Lundqvist C., Boriani G., Castella M., Dan G., Dilaveris P.E. (2020). 2020 ESC Guidelines for the diagnosis and management of atrial fibrillation developed in collaboration with the European Association of Cardio-Thoracic Surgery (EACTS). Eur. Heart J..

[2] Chao T.-F., Tse H.-F., Teo W.-S., Park H.-W., Shimizu W., Chen S.-A., Lip G.Y.H., Asia Pacific Heart Rhythm Society Atrial Fibrillation Registry Investigators (2022). Clinical utility and prognostic implications of the 4S-AF scheme: Report from Asia Pacific Heart Rhythm Society Atrial Fibrillation Registry. Eur. J. Clin. Investig..

[3] Ding W.Y., Proietti M., Boriani G., Fauchier L., Blomström-Lundqvist C., Marin F., Potpara T.S., Lip G.Y.H., ESC-EHRA EORP-AF Long-Term General Registry Investigators (2022). Clinical utility and prognostic implications of the novel 4S-AF scheme to characterize and evaluate patients with atrial fibrillation: A report from ESC-EHRA EORP-AF Long-Term General Registry. Europace.

[4] Potpara T.S., Lip G.Y.H., Blomstrom-Lundqvist C., Boriani G., Van Gelder I.C., Heidbuchel H., Hindricks G., Camm A.J. (2020). The 4S-AF Scheme (Stroke Risk; Symptoms; Severity of Burden; Substrate): A Novel Approach to In-Depth Characterization (Rather than Classification) of Atrial Fibrillation. Thromb. Haemost..

[5] Calkins H., Hindricks G., Cappato R., Kim Y.H., Saad E.B., Aguinaga L., Akar J.G., Badhwar V., Brugada J., Camm J. (2018). 2017 HRS/EHRA/ECAS/APHRS/SOLAECE expert consensus statement on catheter and surgical ablation of atrial fibrillation: Executive summary. Europace.

[6] Mark D.B., Anstrom K.J., Sheng S., Piccini J.P., Baloch K.N., Monahan K.H., Daniels M.R., Bahnson T.D., Poole J.E., Rosenberg Y. (2019). Effect of Catheter Ablation vs Medical Therapy on Quality of Life Among Patients With Atrial Fibrillation: The CABANA Randomized Clinical Trial. JAMA.

[7] Mont L., Bisbal F., Hernández-Madrid A., Pérez-Castellano N., Viñolas X., Arenal A., Arribas F., Lozano I.F., Bodegas A., Cobos A. (2014). Catheter ablation vs. antiarrhythmic drug treatment of persistent atrial fibrillation: A multicentre, randomized, controlled trial (SARA study). Eur. Heart J..

[8] Roy D., Talajic M., Thibault B., Dubuc M., Nattel S., Eisenberg M.J., Ciampi A. (1997). Pilot study and protocol of the Canadian Trial of Atrial Fibrillation (CTAF). Am. J. Cardiol..

[9] Walfridsson H., Walfridsson U., Nielsen J.C., Johannessen A., Raatikainen P., Janzon M., Levin L.A., Aronsson M., Hindricks G., Kongstad O. (2015). Radiofrequency ablation as initial therapy in paroxysmal atrial fibrillation: Results on health-related quality of life and symptom burden. The MANTRA-PAF trial. Europace.

[10] Wazni O.M., Marrouche N.F., Martin D.O., Verma A., Bhargava M., Saliba W., Sterns L.D., Beresh H., Healey J.S., Natale A. (2005). Radiofrequency ablation vs antiarrhythmic drugs as first-line treatment of symptomatic atrial fibrillation: A randomized trial. JAMA.

[11] Jaïs P., Cauchemez B., Macle L., Daoud E., Khairy P., Subbiah R., Hocini M., Extramiana F., Sacher F., Bordachar P. (2008). Catheter ablation versus antiarrhythmic drugs for atrial fibrillation: The A4 study. Circulation.

[12] Wilber D.J., Pappone C., Neuzil P., De Paola A., Marchlinski F., Natale A., Macle L., Daoud E.G., Calkins H., Hall B. (2010). Comparison of antiarrhythmic drug therapy and radiofrequency catheter ablation in patients with paroxysmal atrial fibrillation: A randomized controlled trial. JAMA.

[13] Reynolds M.R., Walczak J., White S.A., Cohen D.J., Wilber D.J. (2010). Improvements in symptoms and quality of life in patients with paroxysmal atrial fibrillation treated with radiofrequency catheter ablation versus antiarrhythmic drugs. Circ. Cardiovasc. Qual. Outcomes.

[14] Blomström-Lundqvist C., Gizurarson S., Schwieler J., Jensen S.M., Bergfeldt L., Kennebäck G., Rubulis A., Malmborg H., Raatikainen P., Lönnerholm S. (2019). Effect of Catheter Ablation vs Antiarrhythmic Medication on Quality of Life in Patients With Atrial Fibrillation: The CAPTAF Randomized Clinical Trial. JAMA.

[15] Morillo C.A., Verma A., Connolly S.J., Kuck K.H., Nair G.M., Champagne J., Sterns L.D., Beresh H., Healey J.S., Natale A. (2014). Radiofrequency ablation vs antiarrhythmic drugs as first-line treatment of paroxysmal atrial fibrillation (RAAFT-2): A randomized trial. JAMA.

[16] Khan M.N., Jaïs P., Cummings J., Di Biase L., Sanders P., Martin D.O., Kautzner J., Hao S., Themistoclakis S., Fanelli R. (2008). Pulmonary-vein isolation for atrial fibrillation in patients with heart failure. N. Engl. J. Med..

[17] Kochhäuser S., Joza J., Essebag V., Proietti R., Koehler J., Tsang B., Wulffhart Z., Pantano A., Khaykin Y., Ziegler P.D. (2016). The Impact of Duration of Atrial Fibrillation Recurrences on Measures of Health-Related Quality of Life and Symptoms. Pacing Clin. Electrophysiol..

[18] Ha A.C., Breithardt G., Camm A.J., Crijns H.J., Fitzmaurice G.M., Kowey P.R., Jle Heuzey E.A., Naditch-Brûlé L., Prystowsky E.N., Schwartz P.J. (2014). Health-related quality of life in patients with atrial fibrillation treated with rhythm control versus rate control: Insights from a prospective international registry (Registry on Cardiac Rhythm Disorders Assessing the Control of Atrial Fibrillation: RECORD-AF). Circ. Cardiovasc. Qual. Outcomes.

[19] Sethi N.J., Feinberg J., Nielsen E.E., Safi S., Gluud C., Jakobsen J.C. (2017). The effects of rhythm control strategies versus rate control strategies for atrial fibrillation and atrial flutter: A systematic review with meta-analysis and Trial Sequential Analysis. PLoS ONE.

[20] Cosedis Nielsen J., Johannessen A., Raatikainen P., Hindricks G., Walfridsson H., Kongstad O., Englund A., Hartikainen J., Mortensen L.S., Hansen P.S. (2012). Radiofrequency ablation as initial therapy in paroxysmal atrial fibrillation. N. Engl. J. Med..

[21] Bulková V., Fiala M., Havránek S., Simek J., Skňouřil L., Januška J., Špinar J., Wichterle D. (2014). Improvement in quality of life after catheter ablation for paroxysmal versus long-standing persistent atrial fibrillation: A prospective study with 3-year follow-up. J. Am. Heart Assoc..

[22] Mohanty S., Santangeli P., Mohanty P., Di Biase L., Holcomb S., Trivedi C., Bai R., Burkhardt D., Hongo R., Hao S. (2014). Catheter ablation of asymptomatic longstanding persistent atrial fibrillation: Impact on quality of life, exercise performance, arrhythmia perception, and arrhythmia-free survival. J. Cardiovasc. Electrophysiol..

[23] Mantovan R., Macle L., De Martino G., Chen J., Morillo C.A., Novak P., Calzolari V., Khaykin Y., Guerra P.G., Nair G. (2013). Relationship of quality of life with procedural success of atrial fibrillation (AF) ablation and postablation AF burden: Substudy of the STAR AF randomized trial. Can. J. Cardiol..

[24] Verma A., Mantovan R., Macle L., De Martino G., Chen J., Morillo C.A., Novak P., Calzolari V., Guerra P.G., Nair G.M. (2010). Substrate and Trigger Ablation for Reduction of Atrial Fibrillation (STAR AF): A randomized, multicentre, international trial. Eur. Heart J..

[25] Pokorney S.D., Kim S., Thomas L., Fonarow G.C., Kowey P.R., Gersh B.J., Mahaffey K.W., Peterson E.D., Piccini J.P. (2017). Cardioversion and subsequent quality of life and natural history of atrial fibrillation. Am. Heart J..

[26] Marrouche N.F., Brachmann J., Andresen D., Siebels J., Boersma L., Jordaens L., Merkely B., Pokushalov E., Sanders P., Proff J. (2018). Catheter Ablation for Atrial Fibrillation with Heart Failure. N. Engl. J. Med..

[27] Verma A., Jiang C-y Betts T.R., Chen J., Deisenhofer I., Mantovan R., Macle L., Morillo C.A., Haverkamp W., Weerasooriya R. (2015). Approaches to Catheter Ablation for Persistent Atrial Fibrillation. N. Engl. J. Med..

[28] Piccini J.P., Todd D.M., Massaro T., Lougee A., Haeusler K.G., Blank B., De Bono J.P., Callans D.J., Elvan A., Fetsch T. (2020). Changes in quality of life, cognition and functional status following catheter ablation of atrial fibrillation. Heart.

[29] Wokhlu A., Monahan K.H., Hodge D.O., Asirvatham S.J., Friedman P.A., Munger T.M., Bradley D.J., Bluhm C.M., Haroldson J.M., Packer D.L. (2010). Long-term quality of life after ablation of atrial fibrillation the impact of recurrence, symptom relief, and placebo effect. J. Am. Coll. Cardiol..

[30] Pontoppidan J., Nielsen J.C., Poulsen S.H., Hansen P.S. (2009). Symptomatic and asymptomatic atrial fibrillation after pulmonary vein ablation and the impact on quality of life. Pacing Clin. Electrophysiol..

[31] Mohanty S., Gianni C., Mohanty P., Halbfass P., Metz T., Trivedi C., Deneke T., Tomassoni G., Bai R., Al-Ahmad A. (2016). Impact of Rotor Ablation in Nonparoxysmal Atrial Fibrillation Patients: Results From the Randomized OASIS Trial. J. Am. Coll. Cardiol..

